# Retraction Note to: Randomized controlled trial testing weight loss and abdominal obesity outcomes of moxibustion

**DOI:** 10.1186/s12938-020-0749-8

**Published:** 2020-01-24

**Authors:** Ching-Hsiu Hsieh, Chi-Chuan Tseng, Ju-Yu Shen, Pei-Ying Chuang

**Affiliations:** 1 Department of Nursing, Chang Gung University of Science and Technology, ChiayiCampus: 2, Chia-pu Rd, West Sec. Pu-tz, 613 Chia-Yi, Taiwan; 2 Department of Traditional Chinese Medicine Chang, Gung Memorial Hospital, Chia-Yi, Taiwan; 3 Department of Early Childhood Educare, Tainan University of Technology, Tainan, Taiwan; 4 School of Nursing, University of Maryland School of Nursing, Baltimore, MD USA

## Retraction to: BioMed Eng OnLine 2018, 17(Suppl 2):149 10.1186/s12938-018-0571-8

The Editors-in-Chief have retracted this article [1] because, after publication, concerns were raised regarding the study design and statistical analysis. Post-publication review has confirmed firstly that within-group changes were highlighted rather than between-group differences as appropriate for a randomized trial, and secondly that there are baseline differences between the groups that exist despite randomization for which analyses were not controlled. The data reported in this article are therefore unreliable. Ching-Hsiu Hsieh and Pei-Ying Chuang agree with this retraction. Chi-Chuan Tseng and Ju-Yu Shen have not responded to any correspondence from the publisher about this retraction.
